# Assessing the role of advanced artificial intelligence as a tool in multidisciplinary tumor board decision-making for recurrent/metastatic head and neck cancer cases – the first study on ChatGPT 4o and a comparison to ChatGPT 4.0

**DOI:** 10.3389/fonc.2024.1455413

**Published:** 2024-09-05

**Authors:** Benedikt Schmidl, Tobias Hütten, Steffi Pigorsch, Fabian Stögbauer, Cosima C. Hoch, Timon Hussain, Barbara Wollenberg, Markus Wirth

**Affiliations:** ^1^ Department of Otolaryngology Head and Neck Surgery, Technical University Munich, Munich, Germany; ^2^ Department of RadioOncology, Technical University Munich, Munich, Germany; ^3^ Institute of Pathology, Technical University Munich, Munich, Germany

**Keywords:** HNSCC, multidisciplinary tumorboard, salvage surgery, artificial intelligence, ChatGPT

## Abstract

**Background:**

Recurrent and metastatic head and neck squamous cell carcinoma (HNSCC) is characterized by a complex therapeutic management that needs to be discussed in multidisciplinary tumor boards (MDT). While artificial intelligence (AI) improved significantly to assist healthcare professionals in making informed treatment decisions for primary cases, an application in the even more complex recurrent/metastatic setting has not been evaluated yet. This study also represents the first evaluation of the recently published LLM ChatGPT 4o, compared to ChatGPT 4.0 for providing therapy recommendations.

**Methods:**

The therapy recommendations for 100 HNSCC cases generated by each LLM, 50 cases of recurrence and 50 cases of distant metastasis were evaluated by two independent reviewers. The primary outcome measured was the quality of the therapy recommendations measured by the following parameters: clinical recommendation, explanation, and summarization.

**Results:**

In this study, ChatGPT 4o and 4.0 provided mostly general answers for surgery, palliative care, or systemic therapy. ChatGPT 4o proved to be 48.5% faster than ChatGPT 4.0. For clinical recommendation, explanation, and summarization both LLMs obtained high scores in terms of performance of therapy recommendations, with no significant differences between both LLMs, but demonstrated to be mostly an assisting tool, requiring validation by an experienced clinician due to a lack of transparency and sometimes recommending treatment modalities that are not part of the current treatment guidelines.

**Conclusion:**

This research demonstrates that ChatGPT 4o and 4.0 share a similar performance, while ChatGPT 4o is significantly faster. Since the current versions cannot tailor therapy recommendations, and sometimes recommend incorrect treatment options and lack information on the source material, advanced AI models at the moment can merely assist in the MDT setting for recurrent/metastatic HNSCC.

## 1 Introduction

Despite recent advancements in immuno-oncology, the five-year survival rate for Head and Neck Squamous Cell Carcinoma (HNSCC) remains poor with approximately 50-60% (1, 2). Recurrence is common in patients with an HNSCC, and the therapy options are limited, resulting in a median overall survival of only 11.8 months (2, 3). Salvage surgery, re-irradiation, and systemic therapies, including cisplatin-based regimens and immunotherapeutic agents, constitute the primary therapeutic options. Additionally, some of the patients already present with distant metastasis at the time of diagnosis limiting the therapy options even further (4). Given that some patients respond well to treatment, while a significant proportion of patients experiences recurrence, each patient is discussed in a multidisciplinary tumor board (MDT) (5, 6). MDTs are essential for providing a multidisciplinary and comprehensive perspective on each case, and for tailoring treatment plans to individual needs (7, 8). On the other hand, MDTs are limited by costs, responsibilities, geographic barriers, and treatment delays (7–9). These limitations have prompted research into artificial intelligence (AI).

AI, in the form of deep learning (DL) and natural language processing (NLP), has opened ways to use Large Language Models (LLMs) like Generative Pre-trained Transformer (GPT) (10, 11) for the MDT setting. While LLMs are constantly evolving, they are able to access large datasets in a short amount of time. Extracting information of recent studies, and the summarization of text are some of the main strengths of LLMs and could potentially be the basis of a modern approach to discuss oncological cases (7–9). This ability to organize and structure data could enable these tools to become an assistance, or even guide MDT-based decision making (10, 12). In the therapeutic and diagnostic setting of HNSCC, ChatGPT achieved an impressive performance in prior studies (12, 13). While most studies of LLMs identified limitations that need to be overcome, including a lack of transparency, the inability to customize therapy recommendations, and sometimes recommending therapy options that do not fully align with established clinical guidelines (12), LLMs are promising tools for enhancing clinical-decision making in the MDT setting of HNSCC. This involves rapidly accessing and summarizing large volumes of clinical information and the latest research, offering evidence-based insights, and streamlining administrative tasks in a time-efficient manner (14). While the evaluation of LLMs lays the foundation of a clinical use in the future, the assessment of the performance is challenging (10). Using validated evaluation tools such as the Artificial Intelligence Performance Instrument (AIPI), is necessary to ensure the reliability, accuracy, and clinical relevance of its recommendations (15).

While prior studies investigated ChatGPT 3.5 and ChatGPT 4.0 for primary HNSCC cases (12, 13, 16, 17), ChatGPT-4 has not yet been evaluated in the decision-making process for recurrent/metastatic Head and Neck Squamous Cell Carcinoma (HNSCC). The novel ChatGPT 4o was just introduced a few weeks ago and is an AI model that is designed to offer enhanced capabilities over its predecessor, ChatGPT 4.0, potentially providing more accurate and contextually appropriate responses. The improvements in ChatGPT 4o include better understanding of more complex queries and improved contextual awareness (18, 19). These advancements suggest that ChatGPT 4o could offer significant benefits over ChatGPT 4.0 and the ability to potentially generate more tailored recommendations. The treatment of such complex cases demands a multidisciplinary approach, thorough knowledge of the latest literature and adherence to evolving clinical guidelines (20), providing a rigorous test of the LLMs capabilities. A comprehensive comparison between the performance of ChatGPT 4.0 and the more recent ChatGPT 4o will be conducted to evaluate the capabilities of ChatGPT 4o in offering therapy recommendations for patients with recurrent/metastatic HNSCC.

## 2 Materials and methods

### 2.1 Patient cohort

This study included patients with a verified recurrent/metastatic HNSCC diagnosis. The electronic patient file and MDT documents provided clinical and histological tumor characteristics before treatment initiation. This study comprised a total of 100 consecutive patients, who have been treated at the Department of Otorhinolaryngology/Head and Neck Surgery, Klinikum rechts der Isar, Technical University of Munich. Recurrent cases were defined as patients who had a local or regional recurrence of HNSCC after initial treatment, with no evidence of distant metastasis at the time of recurrence. Metastatic cases were defined as patients who had distant metastasis beyond the head and neck region. This distinction was made based on imaging studies, histopathological biopsy results, and clinical records. Cases with local recurrence and distal metastasis at the same time were part of the distal metastasis group to differentiate between the two groups and the resulting different therapy options. Out of the patients with recurrence, 76% (38) of the patients had local and regional recurrence, while 12% (6) of the patients had local recurrence and 12% (6) of the patients had regional recurrence in this study. Exclusion criteria included patients with insufficient clinical data or patients who received experimental treatments. To ensure patient confidentiality, the data were anonymized before being shared with the researchers, rendering patient identification impossible. This study was approved by the ethics committee of the Technical University of Munich (Reference: 2024-184_1-S-NP). The characteristics of the patient cohort are depicted in **Table 1**.

**Table 1:** Overview of the patient cohort.

Total patients	100
Sex	
Male	78 (78%)
Female	22 (22%)
**Recurrence**	50
Primary and lymphatic	38(76%)
Primary	6(12%)
Lymphatic	6(12%)
**Distant Metastasis**	50
Subsites	
Larynx	32 (32%)
Oropharynx	28 (28%
Oral Cavity	16(16%
Hypopharynx	10(10%)
Nasal cavity	8(8%
Nasopharynx	4(4%)
Salivary glands	2(2%)
Prior therapy	
Surgery	84(84%)
R(C)Tx	16 (16%)

100 cases of HNSCC were evaluated in this study, with 50 cases of recurrence and 50 cases of distant metastasis. RCTx, radiochemotherapy.

### 2.2 Artificial intelligence/ChatGPT - prompt formatting and data evaluation

ChatGPT 4o and ChatGPT 4.0 are AI chatbots that are accessible to the public. These chatbots use transformer-based language models to generate human-like text responses. The interaction is based on users submitting questions (prompts) through a website interface. The LLMs analyze the contextual relationships between the words in the user’s query to formulate a response. In this study, various prompts were tested, and a standardized prompt format was employed to input patient information into the LLMs, simulating the presentation of an individual patient case in multidisciplinary team (MDT) meetings. Initially, eight prompt variations based on common clinical scenarios in recurrent and metastatic HNSCC were generated and tested with a small subset of 10 randomly selected cases to evaluate responses from both ChatGPT 4o and 4.0. The prompt design mirrored the case presentation format used in MDTs and was iterated several times. The iterations varied in terms of the amount of information and were continuously refined. Two independent reviewers assessed the responses of each prompt for clinical recommendation, explanation, and summarization, rating each on a scale from 1 to 5. The eight prompts and the average total score for the ten cases in the preliminary assessment stage is depicted in the **Supplementary Table 2**. The scales were originally introduced as a tool to evaluate the performance of ChatGPT in the MDT setting of breast cancer (21), but were used in a variety of other studies (22, 23), including the MDT setting of primary HNSCC (12). The tool consists of the three grading scales of summarization, clinical recommendation, and explanation, each ranging from grade 1 (Poor/Total Disagreement) until 5 (Excellent/Total Agreement). The grading scales are further explained and depicted in the Supplementary Material. This pilot scoring phase with multiple iterations led to scorer calibration and training of the two reviewers. The final prompt version reached the highest total score and was selected for providing consistent and accurate therapy recommendations for the main study.

The standard version of the final prompt was “The patient has a history of (XX) for a (XX) carcinoma and now presents with (XX) carcinoma. What treatment options are available and which option do you think leads to the best prognosis?”.

**Figure 1:**
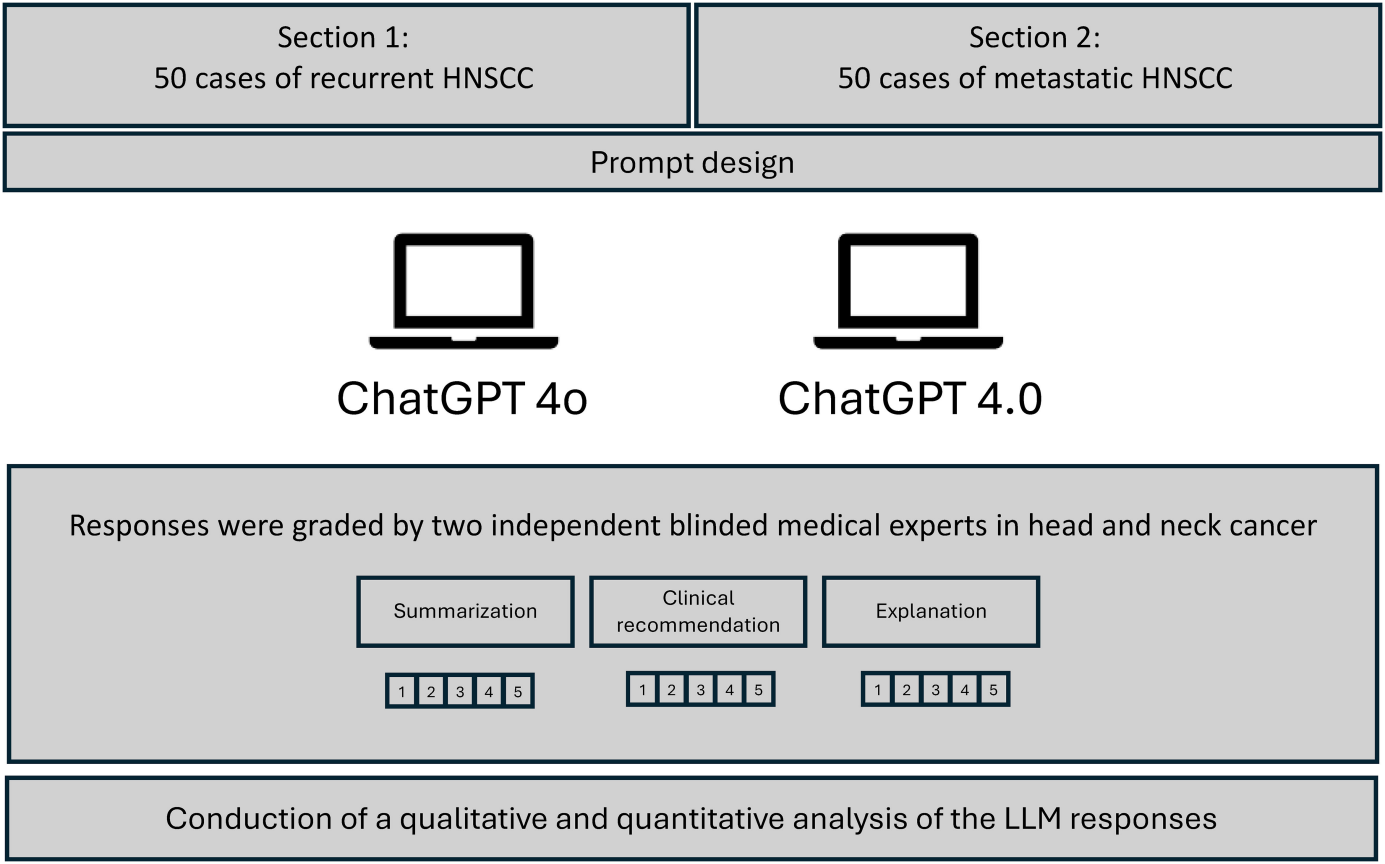
Flowchart of overall study design. Depiction of the grading of responses by ChatGPT 4o and ChatGPT 4.0. Evaluation of the responses by two independent reviewers.

An exemplary prompt was: “The patient has a history of surgery for a cT1a cN0 cM0 glottic laryngeal carcinoma and now presents with a rcT3 rcN1 cM1 glottic laryngeal carcinoma. What treatment options are available and which option do you think leads to the best prognosis?”. No further interaction was initiated after this response; The LLMs prompt history was erased, and the next question was asked. The study design and is shown in a flowchart in **Figure 1**. The responses were collected, and subsequently evaluated using a double-blind method. The two independent reviewers were uninformed about which AI model stated the response. All reviewers independently scored the answers to mitigate subjective biases. The answers provided were assessed using the grading scales for Summarization, Clinical Recommendation, and Explanation, as employed by Sorin et al., 2023 (21). Cohen’s kappa coefficient was used to calculate the inter-rater reliability, providing a measure of the degree to which two raters agree in their categorization of items, corrected for chance agreement. For example, a Kappa value of 0.2 – 0.0 indicates slight agreement, a Kappa value of 0.21 – 0.40 indicates fair agreement, a Kappa value of 0.41 – 0.60 indicates moderate agreement, a Kappa value of 0.61 – 0.80 indicates substantial agreement and a Kappa value of 0.81 – 1.00 indicates perfect agreement between the raters beyond what would be expected by chance. Mann–Whitney U test was used to identify significant differences between the performance of the responses of the two LLMs. A p-value of less than 0.05 was considered statistically significant. P-values were adjusted using the Bonferroni correction method when multiple hypothesis tests were conducted.

## 3 Results

**Figure 2:**
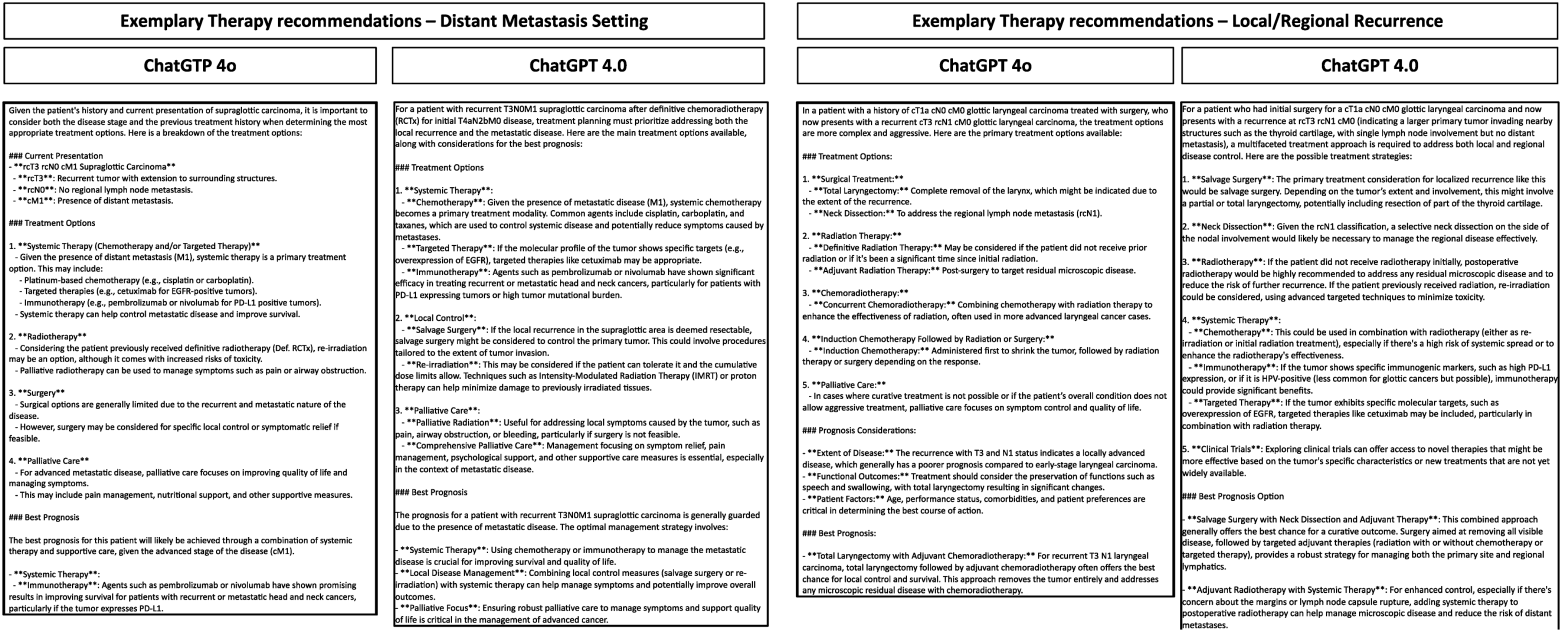
Exemplary prompt and reponses by ChatGPT 4.0 and ChatGPT 4o in the recurrent/metastatic setting of HNSCC. Depicted are the responses of the LLMs for different clinical cases.

ChatGPT 4o and ChatGPT 4.0 answered all prompts in this study in an exceptionally rapid manner. The mean inference speed for ChatGPT 4.0 was 29.7 seconds (± 2.84), while ChatGPT 4o exhibited a mean inference speed of 20.0 seconds (± 5.33), indicating an improved efficiency of 48.5%. For demonstration purposes exemplary responses generated by both LLMs are depicted in **Figure 2**. The design and presentation of the responses varied.

The responses from ChatGPT 4.0 and 4o involved the treatment modalities available for use in the recurrent setting, including salvage surgery and re-irradiation, while in the distant metastatic setting systemic therapy with chemotherapy including cisplatin, carboplatin, and taxanes, targeted therapy, and immunotherapy with pembrolizumab or nivolumab were the most commonly stated answers. Additionally, also in the M1 situation measures of local control were highlighted such as palliative radiation, re-irradiation, salvage surgery, even though this was just recommended for alleviating symptoms such as airway obstruction. Both LLMs had the same primary therapy recommendation in 96% of the M1 cases (48 out of 50). In cases of recurrence, the therapy recommendation was matching in 86% (36 out of 50) of cases. The second choice of therapy was more heterogeneous, with only 38% (19 out of 50) cases matching in the M1 and recurrence situation.

The prognosis of the patients was deemed poor by ChatGPT sometimes even mentioning average five-year survival rates. The clinical history of each case was carefully described by both LLMs using the TNM classification and potential impact of each therapy option. When asked which therapy option leads to the best prognosis, salvage surgery was the most commonly recommended answer of both LLMs in the recurrent setting, but it was stated that this option is only viable for “rare cases where the disease is deemed resectable”. Salvage Neck Dissection was recommended by both LLMs when only lymph node recurrence was present, and in some cases of advanced rcT4b, a surgical approach was not recommended as the preferred treatment option including an explanation of potentially too radical/unfeasible surgery. Metastasis-Directed Surgery such as video-endoscopy assisted thoracoscopy for lung metastasis was mentioned in a few cases.

In both scenarios (recurrence and M1) the importance of multidisciplinary care involving medical oncologists, surgical oncologists, radiation oncologists, and palliative care specialists was highlighted. Especially integrated palliative care was a cornerstone of therapy in almost all cases, with early integration for managing symptoms and improving quality of life rather than curative treatments. Both reviewers mentioned that the LLMs were able to call attention of the presence or lack of specific biomarkers, e.g., PD-L1, EBV DNA levels, to guide the choice of therapy. Important clinical studies for the use of immunotherapy were emphasized.

In only very few cases the therapy recommendation of the LLMs were not according to current guidelines. One of these cases is patient #1, who would have received surgery according to ChatGPT 4o even though the patient already had distant metastasis. Another example is patient #57, who would have received radiochemotherapy for lymph node recurrence by ChatGPT 4o. In case #55 of an rcT1 oral cavity cancer, one of the therapy options of both LLMs was observation and follow-up for a very small, well-differentiated tumor with clear margins post-resection, especially if further radiotherapy is deemed too risky.

**Figure 3:**
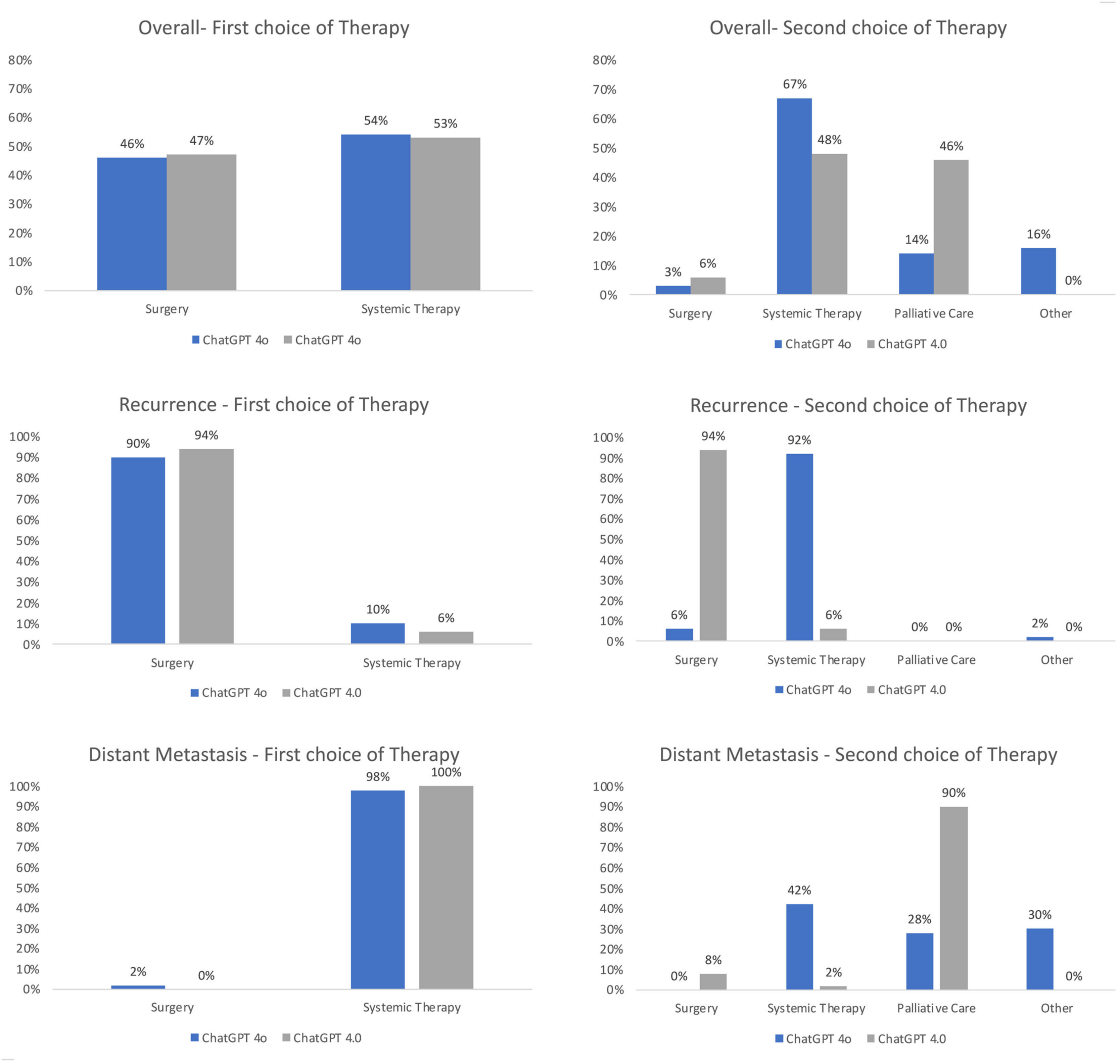
Overview of the recommended treatment options of ChatGPT 4o compared to ChatGPT 4.0. The answers of both LLMs were evaluated by two independent reviewers for the first choice and second choice therapy recommendations in the recurrent and distant metastatic setting. The results were normalized to 100%.

When analyzing the recommended treatment options in detail, one recognizes differences between both LLMs even though the same prompt was used, and the prompt closely resembled the standardized way of presenting a patient at our MDT. An overview of the resulting therapy recommendations is depicted in **Figure 3**. ChatGPT 4o and 4.0 recommended surgery for 90% and 94% of recurrent cases as the first line of therapy, while systemic therapy was only recommended for a few select cases, such as a rcT4b case of oropharyngeal squamous cell carcinoma. Correspondingly, the second choice of therapy was systemic therapy for almost all patients (92% by ChatGPT 4o and 94% by ChatGPT 4.0), with the explicit recommendation of palliative care for one recurrent (rcT4b) case by ChatGPT 4.0. ChatGPT 4o on the other hand recommended the inclusion in a clinical trial for one patient as the second line therapy. In the distant metastatic setting, ChatGPT 4o and 4.0 recommended systemic therapy for 98% and 100% of patients. The second choice of therapy differed between both LLMs, with a recommendation of systemic therapy for 21% of patients by ChatGPT 4o compared to 2% of patients using ChatGPT 4.0, and a recommendation of palliative care for 28% of patients by ChatGPT 4o and 90% of patients by ChatGPT 4.0. Palliative Care was never recommended as a first-choice therapy. Analyzing both the M1 and recurrent setting together, both LLMs recommended similar therapy options, with surgery for 23% vs 23.5% and systemic therapy for 27% vs 26.5% by ChatGPT 4o vs ChatGPT 4.0.

**Figure 4:**
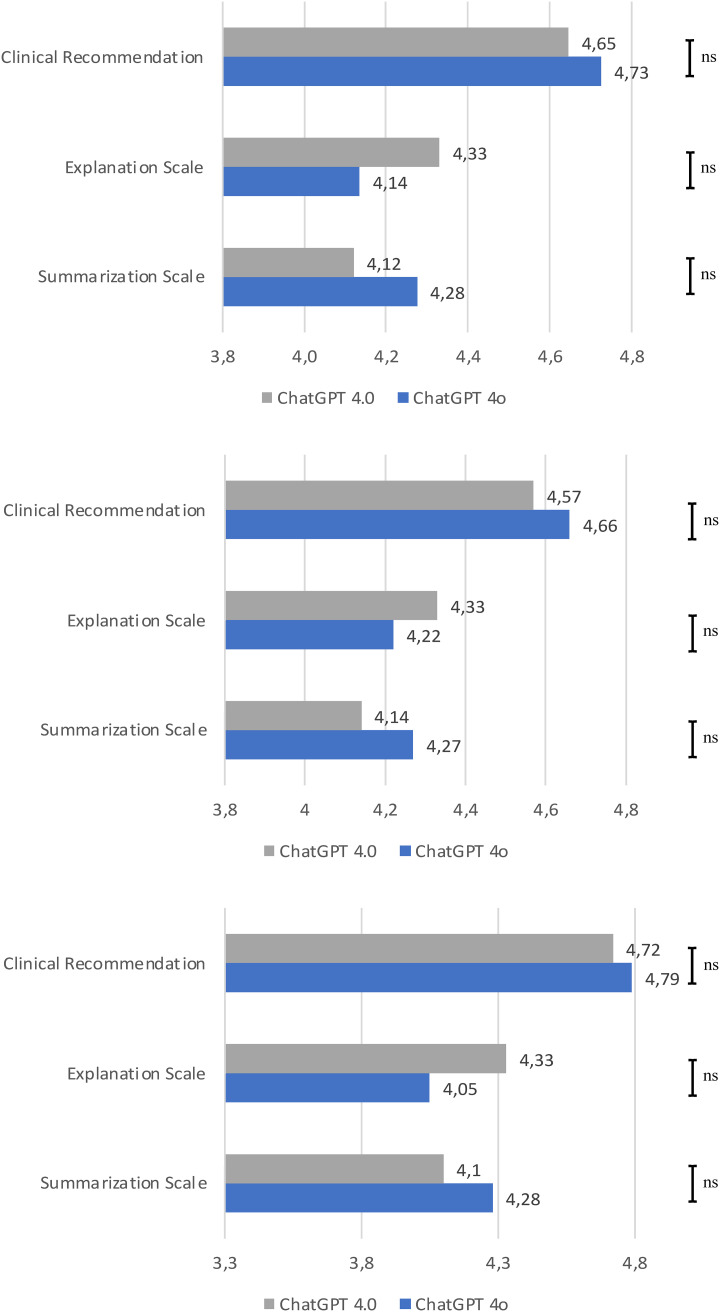
Rating of the performance of ChatGPT 4o and ChatGPT 4.0 by the grading of summarization of text, clinical recommendation, and explanation on the decision made by two independent reviewers. Overall result; Result of the metastatic cases; Result of the recurrent cases. Each bar is the average of the two independent reviewers grading. Statistical significance was calculated using the Mann-Whitney U Test. ns, non significant.

The performance of the LLMs for generating therapy recommendations for recurrent/metastatic HNSCC was evaluated by two independent reviewers. Overall, there was no significant difference in the performance of ChatGPT 4.0 and 4o, with both LLMs reaching similar scores for clinical recommendation (4.65 compared to 4.73, p=0.131), explanation (4.33 compared to 4.14, p=0.423), and summarization (4.12 compared to 4.28, p=0.204). Therefore, ChatGPT 4.0 achieved slightly better results in the explanation grade, while ranking lower in the clinical recommendation and summarization grades (**Figure 4**). In the analysis of recurrent HNSCC, there was also no significant difference in the performance in the grades of clinical recommendation (4.57 compared to 4.66, p=0.362), explanation (4.33 compared to 4.22, p=0.880), and summarization (4.14 compared to 4.27, p=0.200). The same was observed for the distant metastatic cases for clinical recommendation (4.72 compared to 4.79, p=0.214), explanation (4.33 compared to 4.05, p=0.317), and summarization (4.1 compared to 4.28, p=0.657). Clinical recommendation was graded better for cases with distant metastasis compared to the cases with local/regional recurrence. An overview of the statistical analysis is given in the **Supplementary Table 1**. When comparing the recommendations of ChatGPT 4o, two independent reviewers reached an agreement measured by Cohen’s κ of 0.347 for summarization of text, of 0.255 for clinical recommendation, and 0.238 for explanation. When comparing the ChatGPT 4.0 recommendations, two independent reviewers reached an agreement measured by Cohen’s κ of 0.383 for summarization of text, of 0.495 for clinical recommendation, and 0.518 for explanation on the decision made.

**Figure 5:**
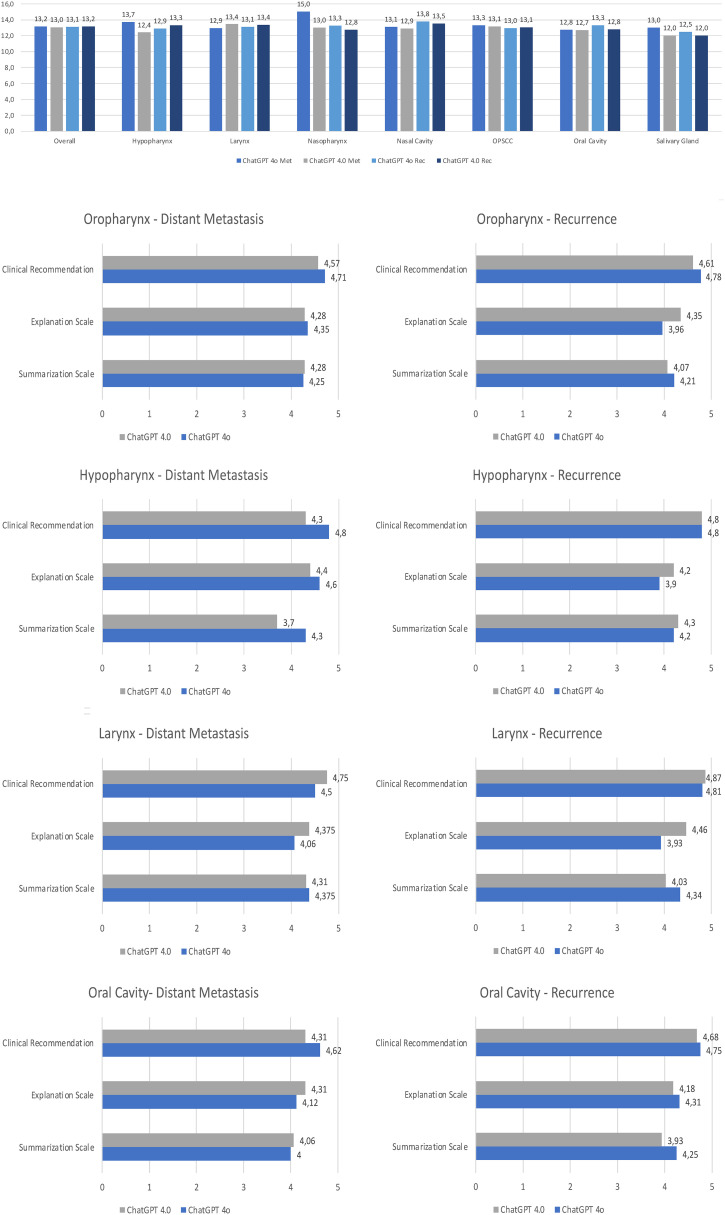
Rating of the performance of ChatGPT 4o and ChatGPT 4.0 according to the setting and the anatomical subsite. Total score of summarization, explanation and clinical recommendation; In depth results of the two LLMs for each subsite. Each bar is the sum of the grading of the two independent reviewers. Met, Metastatic situation; Rec, Recurrence; OPSCC, Oropharyngeal squamous cell carcinoma.

**Figure 6:**
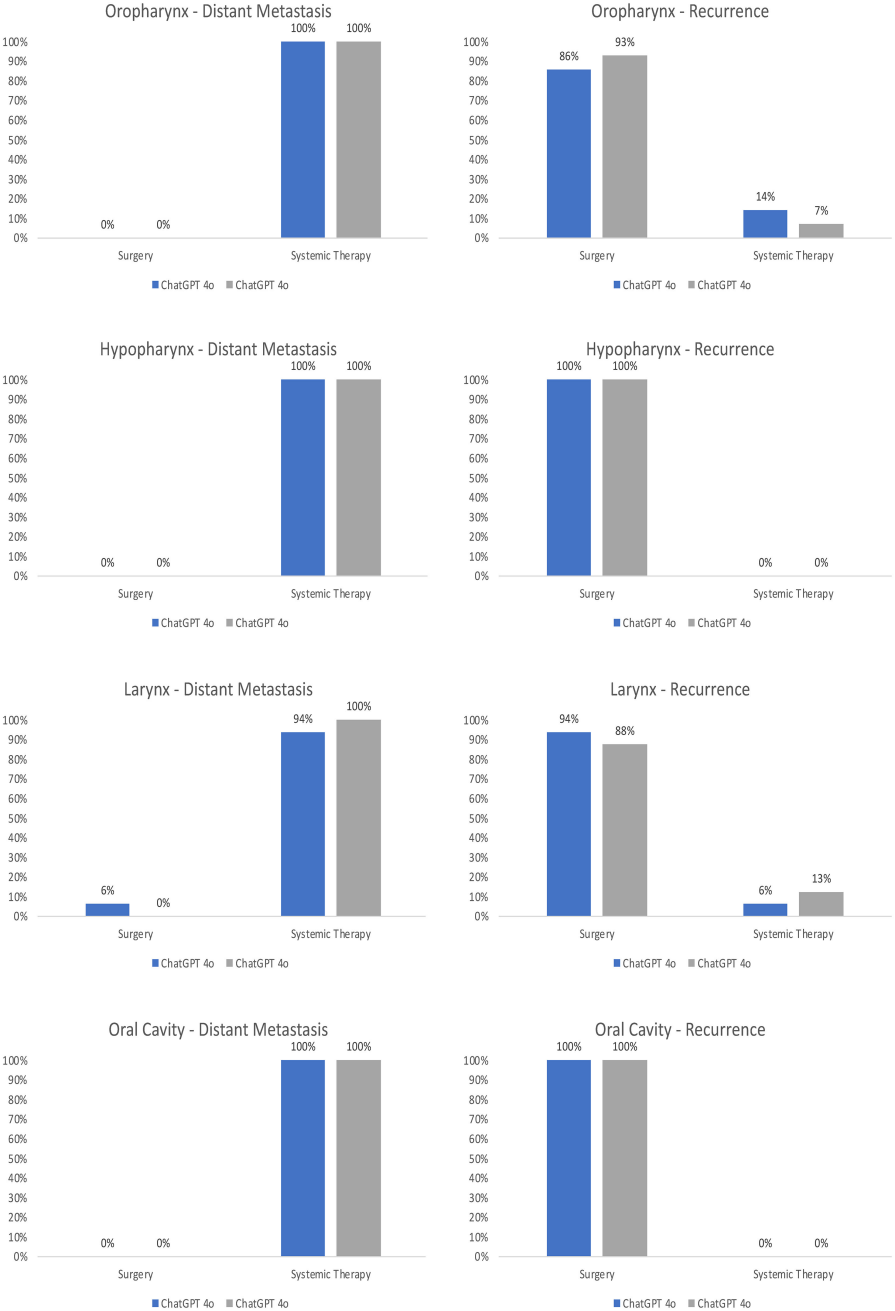
Anatomical subsites of the recommended treatment options of ChatGPT 4o compared to ChatGPT 4.0. The answers of both LLMs were evaluated by two independent reviewers for the first choice and second choice therapy recommendations in the recurrent and distant metastatic setting. The results were normalized to 100%.

The different anatomical subsites out of the 50 cases were also analyzed in detail to explore potential areas of significant expertise (**Figures 5**, **6**). The recommended therapy options differed only slightly between the anatomical subsites. The biggest difference was seen for the local/regional of OPSCC, in which ChatGPT 4o recommended surgery for 86% of the cases, compared to 93% when asked ChatGPT 4.0. For the recurrence of laryngeal cancer ChatGPT 4o recommended surgery for 94% of the cases, compared to 88% when asked ChatGPT 4.0. The results for cancer of the nasal cavity, nasopharynx and salivary glands are depicted in the Supplementary Material. There were also similar results in terms of the qualitative assessment of the overall performance among all subsites without a clear pattern of areas of special expertise, except for a maximum score of ChatGPT 4o for metastatic nasopharyngeal squamous cell carcinoma. This result is limited by the fact that only 4 cases of nasopharyngeal squamous cell carcinoma were included in this study.

Both LLMs refrained from giving precise recommendations, and highlighted, that they are not meant to give medical advice or replace the opinion of a medical doctor. Additionally, both LLMs stated that “Each case should be individually assessed by a multidisciplinary team to tailor the treatment plan according to the patient’s specific disease characteristics, overall health status, and personal preferences, aiming to maximize quality of life and disease control”.

## 4 Discussion

This study represents the first evaluation and comparison of ChatGPT 4o and 4.0 in the currently largest dataset of recurrent/metastatic (R/M) HNSCC. Both LLMs engaged in discussions about the therapy options of these patients, stating potential challenges and the main characteristics of each treatment. The performance for giving therapy recommendations of the LLMs was compared and evaluated by two independent reviewers. The objective of this study was to investigate the potential and limitations of the current landscape of advanced LLMs and to assess a potential use in the multidisciplinary tumor board (MDT) setting. LLMs as a subset of artificial intelligence (AI) focus on the analysis of human language and have found applications across various medical specialties, including head and neck cancer, breast cancer, rheumatology, medical education, and many more (14, 17, 24–27) due to the ability to provide logical and appropriate responses to text questions through the application of advanced language modeling techniques and extensive access to large and diverse datasets (24, 25). Since MDTs have to consider a large quantity of data when reviewing a patient’s case, including the clinical experience, as well as the most recent results of clinical and translational studies (9), LLMs could potentially organize and process data and thereby improve the workflow of clinical decision making. This was also the reason for the first studies investigating the use of AI for the MDT of HNSCC (6, 12, 13). These studies already demonstrated some of the challenges but also potential benefits of using AI for clinical decision making. These studies so far analyzed only a few select recurrent/metastatic HNSCC cases, in some studies even excluding these cases. This study is therefore the first study involving only recurrent/metastatic HNSCC cases, with a total number of 100 patients enrolled. First of all, ChatGPT 4o demonstrated an improved efficiency in terms of processing time with a mean inference speed for ChatGPT 4.0 of 29.7 seconds (± 2.84), while ChatGPT 4o exhibited an improved mean inference speed of 20.0 seconds (± 5.33) can probably be attributed to optimizations in the model’s processing capabilities (19). This is in line with OpenAI promising an up to 50% increased processing speed with ChatGPT 4o compared to ChatGPT 4.0 (28).

At the same time in this study, the performance of ChatGPT 4o was not significantly superior to ChatGPT 4.0 overall, achieving similar and convincing results in the grades of clinical recommendation, explanation, and summarization, with generally ChatGPT 4o being graded slightly better in terms of clinical recommendation, while ChatGPT 4.0 surpassed ChatGPT 4o in the grades of explanation and summarization. At the same time clinical recommendation was graded better for cases with distant metastasis compared to the cases with local/regional recurrence. While there have been no prior study investigating the use ChatGPT 4o for R/M HNSCC, the results of ChatGPT 4.0 are in line with different studies demonstrating an overall convincing performance for oncological decision making, probably due to the ability to access more data than former studies using ChatGPT 3.5 (13, 17, 24). While ChatGPT was already able to access oncological data and provide accurate information about common cancer myths and misconceptions of the National Cancer Institute (29), the performance of more recent ChatGPT versions has not been studied extensively yet (19). According to OpenAI, ChatGPT 4o excels in understanding complex queries and improved contextual awareness, with new functions in the form of audio generation and image recognition (28). For the category of text evaluation the publisher itself lists some of the most commonly performed general benchmark tests, in which ChatGPT 4o reaches similar or slightly improved scores (30). This could explain the results of this study with a similar performance of ChatGPT 4o in comparison to ChatGPT 4.0, since text evaluation might be the critical function of an LLM to assess the clinical setting of recurrent/metastatic HNSCC. Improvements in audio and visual recognition while significant in general applications, might not translate into enhanced performance in the specific and complex environment of recurrent/metastatic HNSCC.

In the next step of this study the subgroups of recurrent and metastatic HNSCC were analyzed separately. The recurrent cases were graded slightly better in terms of clinical recommendation, while this was not significant. Overall, there was no statistically significant difference between both LLMs in the evaluation of recurrent/metastatic HNSCC cases. The in-depth analysis of the anatomical subsites reveals that the results for recurrent cancer of the oropharynx and larynx are the most divergent in terms of therapy recommendations and the performance results. This is probably due to the fact that these subsites are treated differently according to regional guidelines and the therapy is still controversially discussed, as seen in studies on de-escalation therapy of HPV-associated OPSCC or radiotherapy for early glottic cancer (29, 31). Unfortunately, it is currently not possible to investigate the source material leading to the therapy recommendations of the LLMs. While ChatGPT 4o is the latest version of OpenAIs highly performant LLM, and was introduced just a few weeks ago, the main advantages lie in the form of speed, image and audio recognition, cost efficiency, and lastly linguistic comprehension, in which it achieves a slightly better result than ChatGPT 4.0 and Claude 3 Opus (32). Since the MDT setting of recurrent/metastatic cases may not benefit from improvements in speed and linguistic comprehension, this might explain the similar results in this study compared to ChatGPT 4.0. Another potential explanation of the results of this study was the choice of the evaluation method. Even though the tool of Sorin et al. (21) was introduced for evaluating the MDT setting, and was used in different studies and clinical applications including primary HNSCC (12, 22), there are other tools such as the Artificial Intelligence Performance Instrument (AIPI) that have been validated more extensively (15). The AIPI is probably the most validated evaluation tool of ChatGPT so far but was designed primarily for evaluating the diagnostic performance of ChatGPT, containing 9 grades, of which only one grade (#9) evaluates the proposed treatment, therefore limiting the use in studies evaluating the performance of giving therapy recommendations in the MDT setting. Further studies are necessary to establish a validated performance tool in the MDT setting of HNSCC.

In terms of the therapy recommended for the 50 recurrent HNSCC patients, there is also only a slight discrepancy between the LLMs. Both LLMs recommended salvage surgery as the therapy with the best prognosis, and systemic therapy as the second choice. In cases with distant metastasis, systemic therapy was recommended, with a special focus on immunochemotherapy. While in a prior study of the use of ChatGPT 3.5 and 4.0 for the MDT of primary HNSCC (12) immunotherapy was falsely recommended for primary cases, this study demonstrated a more profound knowledge of the indication and approval by the FDA (33–35). Both LLMs are aware of the current guidelines of therapy in the R/M setting and explained the benefits and challenges of each therapy option in a mostly general way. Both LLMs were able to cite some of the most influential studies leading to the current guidelines. Early integration of palliative care was also mentioned by both LLMs, especially by ChatGPT 4.0. This is especially important since head and neck cancer patients have complex palliative care needs and a high degree of symptom burden due to communication issues and other special needs (36). ChatGPT noted the importance of palliative care in the recurrent/metastatic setting of HNSCC, and already suggested many ways health care providers can support people in these difficult situations.

Other studies investigated clinical decision making with ChatGPT for ten consecutive patients with primary breast cancer and compared the treatment recommendations of ChatGPT 3.5 with the MDT and found similarities in 7 out of 10 cases (21). The interrater reliability in this study was similar to the results in our study, demonstrating that the evaluation of the performance of AI tools remains quite subjective. Compared to the results of our study of ChatGPT 4.0 and 4o, prior versions of ChatGPT achieved worse results in terms of decision making for head and neck cancer cases (13).

Since ChatGPT in our study explicitly states that it there has been no prior oncological training (37, 38), which limits the use for tailoring therapy guidelines to the specific needs of the individual patient, other research groups investigated the use of a clinical decision support system based on Bayesian networks (BN) for laryngeal cancer (LC) as a prototype with over 1,000 variables (39–41). In this approach, the TNM classification was the main classifier for the therapeutic recommendations, while ChatGPT 4.0 in this study is able to access an even larger database to address the comorbidities, extent of the tumor and some of the latest studies. Additionally, the software that was used has limited access, and investigated only data of laryngeal carcinoma without data on immunotherapy, therefore restricting the use as a clinical guidance tool in the recurrent/metastatic setting. On the other hand these models are more open and can be programmed and trained by physicians for specific clinical settings, such as the MDT of breast cancer (42), HNSCC (43), or for calculating the survival prognosis of patients (44). These custom programs are often technically demanding and need to be updated and modeled for each new setting, compared to the interactive and intuitive use of an LLMs for a variety of clinical applications without the need for additional programming. Once studies introduce new therapy options or change current guidelines, a Bayesian Network Model needs to be fully revised and trained (43). Since Bayesian Network Models have been trained and validated on medical data, while ChatGPT itself states that it has not received specific medical training, even though the datasets accessed by LLMs involve some of the most recent clinical studies and therefore suggest knowledge in these areas (45), the performance of an LLM for a specific clinical setting needs to be carefully evaluated in studies such as the one in this manuscript.

While the results indicating the quality of recommendations and evaluations for ChatGPT 4o and 4.0 in clinical practice are promising, both large language models have acknowledged certain limitations. Firstly, there is still a lack of transparency of the resources used to answer the prompts, also referred to as the black box of AI. It is unclear how most LLMs arrive at their decision, and it is therefore difficult to understand the rationale behind specific decisions or predictions (46). Therefore, even if all of the recommendations are in line with the current guidelines, the results, must be evaluated carefully due to difficulties in reproduction and validation as seen also in other studies (10, 13, 38). Additionally, LLMs currently lack the level of contextual understanding necessary to customize advice to the unique situation of each patient, one of the most important aspects of personalized medicine and the MDT (12, 47). Patients with recurrent HNSCC often present with a distinct clinical history of previous treatments, genetic factors, and comorbidities. ChatGPT can assist the MDT by integrating patient-specific data with current medical literature and guidelines, but does not possess the ability of tailoring the treatment guidelines onto the specific patient. Without this ability, LLMs are limited to being an assistance and cannot replace the clinical experience of the members of the MDT (13).

Even though this study used a large cohort of 100 patients in the R/M setting, there is most likely a level of heterogeneity due to different anatomical subsites, historical and regional characteristics, which might influence the results of this study (6).

Another limitation is that every answer of an LLM depends heavily on the design of the prompt, with prompt engineering as a new discipline of developing and optimizing prompts to effectively utilize large language models (48). To address this issue, in this study different prompts were tested to find the most accurate prompt to generate convincing answers, while different prompt designs might lead to different responses (16, 25). Due to the potential influence of the prompt design on the performance of an LLM, there have been a few studies and position papers proposing strategies to standardize prompts (49). These prompt strategies include being specific, describing the setting to the LLM, and through testing and iteration (49). In this study the prompt was specifically designed to mirror the way a patient is presented at the MDT and was tested and iterated multiple times to overcome an insufficient performance of ChatGPT due to an error in the prompt design. For this study the same prompt was used both for ChatGPT 4.0 and 4o to allow a direct comparison of the performance in the HNSCC setting, whereas in future studies there might be different prompts for recurrent cases and cases with distant metastasis, since LLMs might need different prompt designs due to currently unknown reasons.

One of the main benefits of the MDT is the ability to discuss the patient’s individual needs in a multidisciplinary setting and facilitate the tailoring of the therapy guidelines to the patient’s situation. This is especially true for the recurrent/metastatic setting, in which the quality of life and the therapy options of a majority of patients are limited due to the side effects of prior radiation or surgical therapy (33, 50). Since LLMs are not able to think independently, generating output based on available public documents and databases (10), they do not possess the ability to tailor individual patient treatment plans. This emphasizes the importance of the MDT and the clinical experience of the health care provider, while the use of AI could potentially improve the efficiency and save time and resources in a period of time, in which the complexity and the number of clinical studies is steadily increasing. Another limitation of our study, is that HNSCC cases of only one central European institution were investigated, whereas all MDTs are influenced by historical, local and personal reasons (7).

Overall, in this study ChatGPT 4o and 4.0 are able to produce convincing answers in terms of summarization, explanation and clinical recommendation for R/M HNSCC in this exploratory study. The performance in terms of overall speed, especially in the case of ChatGPT 4o, can help streamline the decision-making process by providing therapy suggestions and supporting information in seconds. The limitations of the current landscape of LLMs limit the clinical use in the MDT without supervision by an experienced clinician, but the knowledge of advanced LLMs in this study highlights the potential use in the future. Based on the results of this study, a prospective multicenter clinical trial and real-world validation are the next step to rigorously test AI models in the clinical setting of R/M HNSCC to provide robust evidence of their efficacy and safety, ultimately facilitating their integration into clinical practice. The areas of transparency, solid oncological training, as well as ethical concerns need to be addressed to overcome some of the current limitations. Nonetheless, the task of validation and the tailoring of the treatment to the patient will remain in the hands of the MDT and is based on the clinical knowledge of the clinical specialist.

## 5 Conclusions

In this exploratory study, the current version ChatGPT 4o and 4.0 demonstrated a profound knowledge of the indications and treatment options for recurrent and metastatic HNSCC, while there was no significant difference in the performance between both LLMs. Both LLMs achieved convincing grades for explanation, clinical recommendation, and summarization, while ChatGPT 4o was significantly faster than ChatGPT 4.0 in answering the prompts. The current limitations of LLMs demand careful validation and tailoring of the treatment before the implementation into the clinical setting of the MDT.

## Data Availability

The original contributions presented in the study are included in the article/**Supplementary Material**. Further inquiries can be directed to the corresponding author.

## References

[1] Lorini L Bossi P Psyrri A Bonomo P. Human Papilloma Virus (HPV) driven oropharyngeal cancer in current or previous heavy smokers: should we look for a different treatment paradigm? Front Oncol. (2024) 14:1383019. doi: 10.3389/fonc.2024.1383019 38651143 PMC11033308

[2] Johnson DE Burtness B Leemans CR Lui VWY Bauman JE Grandis JR. Head and neck squamous cell carcinoma. Nat Rev Dis Primers. (2020) 6:92–2. doi: 10.1038/s41572-020-00224-3 PMC7944998 33243986

[3] Haring CT Kana LA Dermody SM Brummel C McHugh JB Casper KA. Patterns of recurrence in head and neck squamous cell carcinoma to inform personalized surveillance protocols. Cancer. (2023) 129:2817–27. doi: 10.1002/cncr.34823 37162461

[4] Haddad RI Shin DM. Recent advances in head and neck cancer. New Engl J Med. (2008) 359:1143–54. doi: 10.1056/NEJMra0707975 18784104

[5] Zaboli A Brigo F Sibilio S Mian M Turcato G. Human intelligence versus Chat-GPT: who performs better in correctly classifying patients in triage? Am J Emerg Med. (2024) 79:44–7. 10.1016/j.ajem.2024.02.008 38341993

[6] Lechien JR Chiesa-Estomba CM Baudouin R Hans S. Accuracy of ChatGPT in head and neck oncological board decisions: preliminary findings. Eur Arch Otorhinolaryngol. (2024) 281:2105–14. doi: 10.1007/s00405-023-08326-w 37991498

[7] Berardi R Morgese F Rinaldi S Torniai M Mentrasti G Scortichini L. Benefits and limitations of a multidisciplinary approach in cancer patient management. Cancer Manag Res. (2020) 12:9363–74. doi: 10.2147/CMAR.S220976 PMC7533227 33061625

[8] Thenappan A Halaweish I Mody RJ Smith EA Geiger JD Ehrlich PF. Review at a multidisciplinary tumor board impacts critical management decisions of pediatric patients with cancer. Pediatr Blood Cancer. (2017) 64:254–8. doi: 10.1002/pbc.v64.2 27578484

[9] Luchini C Lawlor RT Milella M Scarpa A. Molecular tumor boards in clinical practice. Trends Cancer. (2020) 6:738–44. doi: 10.1016/j.trecan.2020.05.008 32517959

[10] Cascella M Montomoli J Bellini V Bignami E. Evaluating the feasibility of chatGPT in healthcare: an analysis of multiple clinical and research scenarios. J Med Syst. (2023) 47:33. doi: 10.1007/s10916-023-01925-4 36869927 PMC9985086

[11] Sufi F. Generative pre-trained transformer (GPT) in research: A systematic review on data augmentation. Information. (2024) 15:99. doi: 10.3390/info15020099

[12] Schmidl B Hütten T Pigorsch S Stögbauer F Hoch CC Hussain T. Assessing the role of advanced artificial intelligence as a tool in multidisciplinary tumor board decision-making for primary head and neck cancer cases. Front Oncol. (2024) 14. doi: 10.3389/fonc.2024.1353031 PMC11157509 38854718

[13] Lechien JR Georgescu BM Hans S Chiesa-Estomba CM. ChatGPT performance in laryngology and head and neck surgery: a clinical case-series. Eur Arch Otorhinolaryngol. (2023). doi: 10.1007/s00405-023-08282-5 37874336

[14] Lukac S Dayan D Fink V Leinert E Hartkopf A Veselinovic K. Evaluating ChatGPT as an adjunct for the multidisciplinary tumor board decision-making in primary breast cancer cases. Arch Gynecol Obstet. (2023) 308:1831–44. doi: 10.1007/s00404-023-07130-5 PMC10579162 37458761

[15] Lechien JR Maniaci A Gengler I Hans S Chiesa-Estomba CM Vaira LA. Validity and reliability of an instrument evaluating the performance of intelligent chatbot: the Artificial Intelligence Performance Instrument (AIPI). Eur Arch Otorhinolaryngol. (2024) 281:2063–79. doi: 10.1007/s00405-023-08219-y 37698703

[16] Benary M Wang XD Schmidt M Soll D Hilfenhaus G Nassir M. Leveraging large language models for decision support in personalized oncology. JAMA Netw Open. (2023) 6:e2343689. doi: 10.1001/jamanetworkopen.2023.43689 37976064 PMC10656647

[17] Huang Y Gomaa A Semrau S Haderlein M Lettmaier S Weissmann T. Benchmarking ChatGPT-4 on a radiation oncology in-training exam and Red Journal Gray Zone cases: potentials and challenges for ai-assisted medical education and decision making in radiation oncology. Front Oncol. (2023) 13:1265024. doi: 10.3389/fonc.2023.1265024 37790756 PMC10543650

[18] Mu LJ Wang TT Miao YD. Advancements in AI-driven oncology: assessing chatGPT’s impact from GPT-3.5 to GPT-4o. Int J Surg. (2024). doi: 10.1097/JS9.0000000000001989 PMC11745765 39041947

[19] Zhang N Sun Z Xie Y Wu H Li C. The latest version ChatGPT powered by GPT-4o: what will it bring to the medical field? Int J Surg. (2024). doi: 10.1097/JS9.0000000000001754 PMC11392067 38857508

[20] Deng L Yangzhang Wang T Zhai Z Tao W Li J. Evaluation of large language models in breast cancer clinical scenarios: a comparative analysis based on ChatGPT-3.5, ChatGPT-4.0, and Claude2. Int J Surg. (2024) 110:1941–50. doi: 10.1097/JS9.0000000000001066 PMC11019981 38668655

[21] Sorin V Klang E Sklair-Levy M Cohen I Zippel DB Balint Lahat N. Large language model (ChatGPT) as a support tool for breast tumor board. NPJ Breast Cancer. (2023) 9:44. doi: 10.1038/s41523-023-00557-8 37253791 PMC10229606

[22] Stalp JL Denecke A Jentschke M Hillemanns P Klapdor R. Quality of chatGPT-generated therapy recommendations for breast cancer treatment in gynecology. Curr Oncol. (2024) 31:3845–54. doi: 10.3390/curroncol31070284 PMC11275284 39057156

[23] Frosolini A Catarzi L Benedetti S Latini L Chisci G Franz L. The role of large language models (LLMs) in providing triage for maxillofacial trauma cases: A preliminary study. Diagnostics. (2024) 14:839. doi: 10.3390/diagnostics14080839 38667484 PMC11048758

[24] Hughes KS Zhou J Bao Y Singh P Wang J Yin K. Natural language processing to facilitate breast cancer research and management. Breast J. (2020) 26:92–9. doi: 10.1111/tbj.13718 31854067

[25] Hügle T. The wide range of opportunities for large language models such as ChatGPT in rheumatology. RMD Open. (2023) 9. doi: 10.1136/rmdopen-2023-003105 PMC10151992 37116985

[26] Kanjee Z Crowe B Rodman A. Accuracy of a generative artificial intelligence model in a complex diagnostic challenge. JAMA. (2023) 330:78–80. doi: 10.1001/jama.2023.8288 37318797 PMC10273128

[27] Chakraborty C Pal S Bhattacharya M Dash S Lee S-S. Overview of Chatbots with special emphasis on artificial intelligence-enabled ChatGPT in medical science. Front Artif Intell. (2023) 6. doi: 10.3389/frai.2023.1237704 PMC10644239 38028668

[28] Leo Ueno TL. GPT-4o: The Comprehensive Guide and Explanation. (2024).

[29] Johnson SB King AJ Warner EL Aneja S Kann BH Bylund CL. Using ChatGPT to evaluate cancer myths and misconceptions: artificial intelligence and cancer information. JNCI Cancer Spectr. (2023) 7. doi: 10.1093/jncics/pkad015 PMC10020140 36929393

[30] OpenAI. Image inputs for ChatGPT - FAQ. (2024).

[31] Benchetrit L Torabi SJ Givi B Haughey B Judson BL. Prognostic significance of extranodal extension in HPV-mediated oropharyngeal carcinoma: A systematic review and meta-analysis. Otolaryngol Head Neck Surg. (2021) 164:720–32. doi: 10.1177/0194599820951176 32838649

[32] Montañés A. What are the differences between ChatGPT-4 and ChatGPT-4o? RAONA (2024).

[33] Cohen EE LaMonte SJ Erb NL Beckman KL Sadeghi N Hutcheson KA. American cancer society head and neck cancer survivorship care guideline. CA Cancer J Clin. (2016) 66:203–39. doi: 10.3322/caac.21343 27002678

[34] Hanna GJ O'Neill A Shin KY Wong K Jo VY Quinn CT. Neoadjuvant and adjuvant nivolumab and lirilumab in patients with recurrent, resectable squamous cell carcinoma of the head and neck. Clin Cancer Res. (2022) 28:468–78. doi: 10.1158/1078-0432.CCR-21-2635 PMC9401515 34667025

[35] Hanna GJ Villa A Shi R O'Neill A Liu M Quinn CT. 650O A phase II study of nivolumab for high-risk oral leukoplakia. Ann Oncol. (2022) 33:S839. doi: 10.1016/j.annonc.2022.07.774

[36] Mayland CR Ho QM Doughty HC Rogers SN Peddinti P Chada P. The palliative care needs and experiences of people with advanced head and neck cancer: A scoping review. Palliat Med. (2021) 35:27–44. doi: 10.1177/0269216320963892 33084497 PMC7797618

[37] Temsah O Khan SA Chaiah Y Senjab A Alhasan K Jamal A. Overview of early chatGPT’s presence in medical literature: insights from a hybrid literature review by chatGPT and human experts. Cureus. (2023) 15:e37281. doi: 10.7759/cureus.37281 37038381 PMC10082551

[38] Uprety D Zhu D West HJ. ChatGPT-A promising generative AI tool and its implications for cancer care. Cancer. (2023) 129:2284–9. doi: 10.1002/cncr.34827 37183438

[39] Gaebel J Wu HG Oeser A Cypko MA Stoehr M Dietz A. Modeling and processing up-to-dateness of patient information in probabilistic therapy decision support. Artif Intell Med. (2020) 104:101842. doi: 10.1016/j.artmed.2020.101842 32499009

[40] Hikal A Gaebel J Neumuth T Dietz A Stoehr M. A treatment decision support model for laryngeal cancer based on bayesian networks. Biomedicines. (2023) 11. doi: 10.3390/biomedicines11010110 PMC9855792 36672618

[41] Cypko MA Stoehr M Oeltze-Jafra S Dietz A Lemke HU. A guide for constructing bayesian network graphs of cancer treatment decisions. Stud Health Technol Inform. (2017) 245:1355. 29295434

[42] Schettini F Venturini S Giuliano M Lambertini M Pinato DJ Onesti CE. Multiple Bayesian network meta-analyses to establish therapeutic algorithms for metastatic triple negative breast cancer. Cancer Treat Rev. (2022) 111:102468. doi: 10.1016/j.ctrv.2022.102468 36202026

[43] Cypko MA Stoehr M Kozniewski M Druzdzel MJ Dietz A Berliner L. Validation workflow for a clinical Bayesian network model in multidisciplinary decision making in head and neck oncology treatment. Int J Comput Assist Radiol Surg. (2017) 12:1959–70. doi: 10.1007/s11548-017-1531-7 28204986

[44] Zhang Z Chai H Wang Y Pan Z Yang Y. Cancer survival prognosis with Deep Bayesian Perturbation Cox Network. Comput Biol Med. (2022) 141:105012. doi: 10.1016/j.compbiomed.2021.105012 34785075

[45] Dave T Athaluri SA Singh S. ChatGPT in medicine: an overview of its applications, advantages, limitations, future prospects, and ethical considerations. Front Artif Intell. (2023) 6. doi: 10.3389/frai.2023.1169595 PMC10192861 37215063

[46] Gallistl V Banday MUL Berridge C Grigorovich A Jarke J Mannheim I. Addressing the black box of AI - A model and research agenda on the co-constitution of aging and artificial intelligence. Gerontologist. (2024). doi: 10.1093/geront/gnae039 PMC11134299 38700416

[47] Goetz LH Schork NJ. Personalized medicine: motivation, challenges, and progress. Fertil Steril. (2018) 109:952–63. doi: 10.1016/j.fertnstert.2018.05.006 PMC6366451 29935653

[48] Giray L. Prompt engineering with chatGPT: A guide for academic writers. Ann BioMed Eng. (2023) 51:2629–33. doi: 10.1007/s10439-023-03272-4 37284994

[49] Wang J Shi E Yu S Wu Z Ma C Dai H. Prompt engineering for healthcare: Methodologies and applications. (2023).

[50] Cohen EEW Bell RB Bifulco CB Burtness B Gillison ML Harrington KJ. The Society for Immunotherapy of Cancer consensus statement on immunotherapy for the treatment of squamous cell carcinoma of the head and neck (HNSCC). J Immunother Cancer. (2019) 7:184–4. doi: 10.1186/s40425-019-0662-5 PMC6632213 31307547

