# Supplementary material for: Assessing the role of advanced artificial intelligence as a tool in multidisciplinary tumor board decision-making for recurrent/metastatic head and neck cancer cases – the first study on ChatGPT 4o and a comparison to ChatGPT 4.0
Source: Front Oncol. 2024 Sep 5;14:1455413. doi: 10.3389/fonc.2024.1455413 (PMC11410764; doi:10.3389/fonc.2024.1455413)
Supplement: Supplementary file 1 [file DataSheet1.docx]

**Assessing the role of advanced artificial intelligence as a tool in multidisciplinary tumor board decision-making for recurrent/metastatic head and neck cancer cases – The first study on ChatGPT 4o and a comparison to ChatGPT 4**

**Supplementary Material**

**Supplementary Fig 1: Grading Scales (Sorin et al. 2023): Summarization**

| Grade | Definition |
| --- | --- |
| 1 - Poor (Total Disagreement) | The algorithm-generated summary fails to capture the essential information from the clinical data. It may contain inaccuracies or irrelevant information. |
| 2 - Below Average (Disagreement) | The summary captures some important points from the clinical data, but misses other key aspects. It may contain minor inaccuracies or redundancies. |
| 3 - Average (Neutrality) | The summary adequately represents the key points of the clinical data. It is generally correct and comprehensible, but could be improved in terms of clarity or conciseness. |
| 4 - Above Average (Agreement) | The summary effectively captures the important information from the clinical data. It is accurate and clear, requiring minimal improvements. |
| 5 – Excellent (Total Agreement) | The algorithm-generated summary perfectly captures the essential information from the clinical data. It is highly accurate, clear, and concise, leaving no room for improvement. |

**Grading Scales: Clinical Recommendation**

| Grade | Definition |
| --- | --- |
| 1 - Poor (Total Disagreement) | The algorithm's treatment recommendation is entirely incorrect or potentially harmful for the patient. It may have misinterpreted or ignored critical clinical data in the decision-making process. |
| 2 - Below Average (Disagreement) | The algorithm's treatment recommendation is not ideal or suboptimal for the patient's condition. It may have considered some relevant clinical data but failed to account for other important factors. |
| 3 - Average (Neutrality) | The algorithm's treatment recommendation is generally appropriate for the patient's condition. It has considered most of the relevant clinical data and has arrived at a treatment decision that a physician might also recommend. |
| 4 - Above Average (Agreement) | The algorithm's treatment recommendation is well-suited for the patient's condition. It has taken into account all relevant clinical data and has arrived at a treatment decision that is aligned with an expert (breast oncologist/breast surgeon) recommendation. |
| 5 – Excellent (Total Agreement) | The algorithm's treatment recommendation is optimal for the patient's condition. It has thoroughly analyzed all relevant clinical data and provided a treatment decision that demonstrates a deep understanding of the patient's needs, and aligns with an expert (creast oncologist/breast surgeon) recommendation. |

**Grading Scales: Explanation**

| Grade | Definition |
| --- | --- |
| 1 - Poor (Total Disagreement) | The algorithm's explanation for the clinical situation and its decision-making process is unclear, confusing, or incorrect. It may contain inaccuracies or irrelevant information. |
| 2 - Below Average (Disagreement) | The explanation provides some insight into the algorithm's decision-making process, but may lack clarity or coherence. It may contain inaccuracies or lack important details. |
| 3 - Average (Neutrality) | The explanation is generally clear and understandable, but could be improved in terms of detail or organization. |
| 4 - Above Average (Agreement) | The explanation effectively describes the patient’s clinical status and the algorithm's decision-making process. It is clear, coherent, and well-structured, with minimal room for improvement. |
| 5 – Excellent (Total Agreement) | The algorithm's explanation is accurate, and easy to understand. It thoroughly demonstrates the reasoning behind the decision-making process, leaving no room for improvement. |

**Supplementary Table 1: Statistical Analysis of the Comparison of the performance of ChatGPT 4o vs 4.0**

**Supplementary Table 2: Prompt design and iterations for assessing the performance of ChatGPT 4o vs 4.0 for R/M HNSCC**

| # | Prompt Design | Average Total Score of the Performance (10 cases) |
| --- | --- | --- |
| Prompt 1 | The patient presents with (XX) carcinoma. Which therapy option leads to the best outcome?” | 9,8 |
| Prompt 2 | The patient presents with (XX) carcinoma. What treatment options are available and which option leads to the best outcome?” | 10,5 |
| Prompt 3 | The patient presents with (XX) carcinoma. What treatment options are available and which option do you think leads to the best prognosis?” | 12,2 |
| Prompt 4 | The patient has a history of (XX) for a (XX) carcinoma and now presents with (XX) carcinoma. Which treatment options lead to the best prognosis?” | 11,7 |
| Prompt 5 | The patient has a history of (XX) for a (XX) carcinoma and now presents with (XX) carcinoma. The patient is (XX) years old, is (XX) and has the following comorbidities (XX). Which treatment do you think leads to the best prognosis?” | 12,1 |
| Prompt 6 | The patient has a history of (XX) for a (XX) carcinoma and now presents with (XX) carcinoma. The patient has the following comorbidities (XX). Which treatment do you think leads to the best prognosis?” | 13.1 |
| Prompt 7 | The patient has a history of (XX) for a (XX) carcinoma and now presents with (XX) carcinoma. What treatment options are available and which option do you think leads to the best prognosis?” | 13,4 |
| Prompt 8 | The patient has a history of (XX) for a (XX) carcinoma and now presents with (XX) carcinoma. The patient is (XX) years old, is (XX) and has the following comorbidities (XX). In the blood there was (XX). The patient had a BMI of (XX) and had the following risk behavior (XX). Which treatment do you think leads to the best prognosis?” | 12,6 |
